# Larger Right Posterior Parietal Volume in Action Video Game Experts: A Behavioral and Voxel-Based Morphometry (VBM) Study

**DOI:** 10.1371/journal.pone.0066998

**Published:** 2013-06-11

**Authors:** Satoshi Tanaka, Hanako Ikeda, Kazumi Kasahara, Ryo Kato, Hiroyuki Tsubomi, Sho K. Sugawara, Makoto Mori, Takashi Hanakawa, Norihiro Sadato, Manabu Honda, Katsumi Watanabe

**Affiliations:** 1 Nagoya Institute of Technology, Aichi, Japan; 2 Rikkyo University, Tokyo, Japan; 3 Japan Society for Promotion of Science, Tokyo, Japan; 4 National Center of Neurology and Psychiatry, Tokyo, Japan; 5 Waseda University, Tokyo, Japan; 6 Toyama University, Toyama, Japan; 7 National Institute for Physiological Sciences, Aichi, Japan; 8 Chiba Prefectural Board of Education, Chiba, Japan; 9 Japan Science and Technology Agency, Saitama, Japan; 10 The University of Tokyo, Tokyo, Japan; University of Manchester, United Kingdom

## Abstract

Recent studies suggest that action video game players exhibit superior performance in visuospatial cognitive tasks compared with non-game players. However, the neural basis underlying this visuospatial cognitive performance advantage remains largely unknown. The present human behavioral and imaging study compared gray matter volume in action video game experts and non-experts using structural magnetic resonance imaging and voxel-based morphometry analysis. The results revealed significantly larger gray matter volume in the right posterior parietal cortex in experts compared with non-experts. Furthermore, the larger gray matter volume in the right posterior parietal cortex significantly correlated with individual performance in a visual working memory task in experts. These results suggest that differences in brain structure may be linked to extensive video game play, leading to superior visuospatial cognitive performance in action video game experts.

## Introduction

With recent developments in Internet-based games and portable game devices, video games have become increasingly accessible in daily life. Recent studies suggest that video game playing improves a range of human visuospatial cognitive abilities [1], [2]. So far, many studies examining visuospatial cognition have focused on one specific genre of games; action video games (AVG), which emphasize visuospatial and physical challenges [2]. A large body of behavioral evidence suggests that AVG players exhibit superior performance in a variety of untrained, visuospatial cognitive tasks, such as those that require spatial attention [3]–[13], mental rotation [14], [15], working memory [15], [16] and visuomotor skills [17]. While there is controversy about whether AVGs improve performance in untrained tasks [18]–[20], these studies have increased interest in the educational and rehabilitative potential of AVGs for improving cognitive, perceptual, and motor function. [8], [15], [21].

At present, only a few studies have investigated possible differences between AVG players and non-players at the neural level, or the neural basis for increased performance in visuospatial cognitive tasks [22]–[25]. The present study examined differences in gray matter (GM) volume between AVG players and non-players using high-resolution anatomical magnetic resonance imaging (MRI) and voxel-based morphometry (VBM) analysis [26]. Recent cross-sectional studies have reported differences in region-specific GM volume between individuals who hold expert and non-expert status in other cognitive and motor activities [27]–[32]. Thus, to maximize the possible contrast between AVG players and non-players, the present study examined highly experienced and skilled AVG experts, who had won several prizes at AVG competitions.

A large body of neuroimaging evidence suggests that the posterior parietal cortex (PPC) is involved in visuospatial cognitive functions, such as attention, working memory (WM), and visual imagery [33]–[38]. PPC lesions, particularly in the right hemisphere, can induce visual neglect and a variety of visuospatial cognitive functional deficits [35], [39]. Therefore, we hypothesized that [(1)] GM volume in the PPC of AVG experts would be larger than in non-experts, and [(2)] larger GM volume in the PPC of AVG experts would correlate with increased performance in a visuospatial cognitive task.

## Methods

### Participants

A total of 50 right-handed males participated in the study. Seventeen participants were expert AVG players (game expert group; mean age 24.1 years, SD = 2.9). The experts had, on average, 15.9 years (SD = 4.5) of extensive experience in video game play, and the mean age of video-game commencement was 8.1 years (SD = 3.5). Individuals in this group played AVGs for approximately 20 hours per week at the time of participation in the study (mean 21.4 hours, SD = 10.0). All expert participants had received several prizes at AVG competitions. For example, 16/17 participants in the game expert group were ranked within the top 2–32 at the world's largest AVG competition (SUPER BATTLE OPERA: Arcadia cup tournament, involving approximately 25,000 game players). The remaining participant had won a tournament in a well-known domestic AVG competition. Therefore, the expert group was relatively homogeneous in terms of gaming experience. The AVG expert group all played *Guilty Gear* (Arc System Works, Yokohama, Japan) in the Arcadia cup tournament. *Guilty Gear* is a competitive third-person fighting game, which is a subgenre of AVGs. In the game display, two on-screen characters face off in close one-on-one combat. Players can perform basic attacks like kicking and banging using simple actions such as pressing a single button. Stronger and more efficient attacks require more complex combinations of button presses and lever movements. When an opponent character is controlled by a human player (not by computer), opponent characters and their weapons (e.g., swords, bullets or laser beams) often move very quickly and unpredictably. Therefore, in addition to well-developed visuo-motor skills, the game involves substantial attentional focus. Thus, *Guilty Gear* has similar cognitive requirements to the shooter games used in previous AVG studies (e.g., [3]).

The other 33 participants had negligible or no video game experience (i.e. less than two hours of video game play per week) and served as the control group (mean age 22.4 years, SD = 3.42). There was no significant age difference between the expert and control groups (*t* [(48)] = 0.90, *p* = 0.37). Educational level was matched between the groups, as all participants' education was undergraduate level. None of the participants had a history of psychiatric or neurological illness. The experiment was approved by the local ethics committee of the National Center of Neurology and Psychiatry in Japan. Written informed consent was obtained from all participants prior to testing. All participants were male, because of difficulties in recruiting female participants with sufficient video game experience.

### Visual Working Memory Task

**Figure 1:**
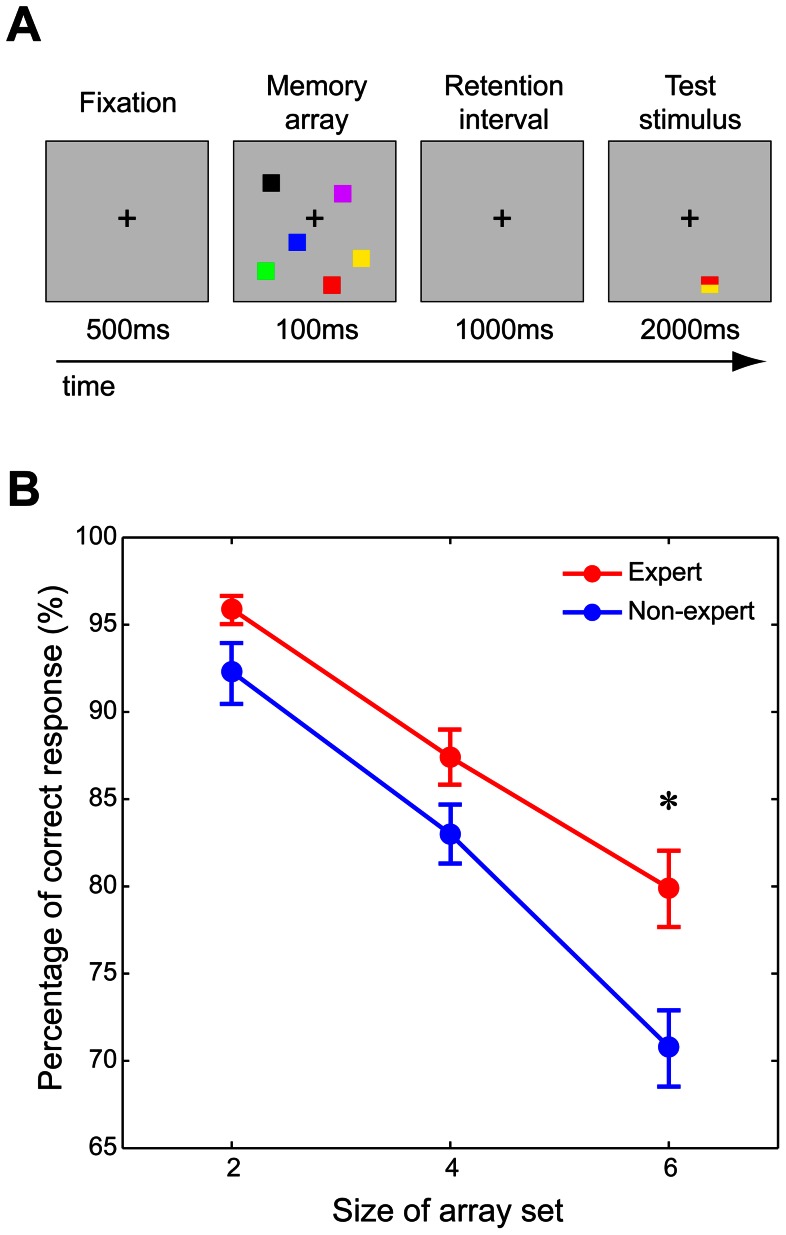
Visual working memory (WM) task. A: Experimental paradigms of the visual WM task. First, a sample array was presented for 100 ms on the computer display. Each sample array consisted of two, four, or six colored squares at randomized positions (in the present figure, the size of sample array is 6). After a 1,000-ms retention interval, the test stimulus was presented for 2,000ms at one of the sample array positions. Each test cue consisted of two colored rectangles that were half the width of the sample squares. Participates were asked to determine which test stimulus color was the same as the sample square that had been shown at that position. B: Behavioral results from the visual WM task. Data are presented as the group mean of percentage of correct responses, with bars indicating standard errors. The red and blue circles indicate data from AVG experts and non-experts, respectively. The horizontal axis represents sample array size. The percentage of correct responses in AVG experts is significantly greater than in the non-experts in the task with an array size of six. * indicates *p*<0.05 after correction for multiple comparisons (Bonferroni correction).

We used a modified version of the visual WM task described by Luck and Vogel [40] as our behavioral task (Fig. 1A). In most AVGs, multiple objects are simultaneously presented in the visual field, and players must rapidly memorize them to search for useful visual information. Successful performance primarily relies on the visual WM capacity of a player, which suggests that AVG players are likely to possess greater visual WM capacity than AVG non-players.

Presentation software (Neurobehavioral Systems, CA, USA) was used for visual stimulus presentation and for recording participant responses. After presentation of a visual fixation cross at the center of the computer screen, a sample array appeared for 100 ms. This was followed by a 1,000-ms retention interval and then a test stimulus. The test stimulus was presented for 2,000 ms. All sample arrays were presented within 12.4°×11.3° rectangular regions on a grey background. Each sample array consisted of two, four, or six colored squares (1.6°×1.6°). The color of each square was randomly selected from a set of nine clearly discriminable colors (red, brown, blue, cyan, violet, green, yellow, black, and white), and each color appeared only once in an array. The positions of the squares were randomized for each trial, with the constraint that the distance between squares was at least 2.6° (center to center). The test stimulus was presented at one of the square positions in the sample array. Each test stimulus consisted of two colored rectangles that were half the width of the sample squares. One rectangle was the same color as the sample square that had been at that position, and the other was a color that was different than that of the previous square. Participants were asked to report, by a button press, whether a color in the test stimulus was the same color as the sample square that had been at that position.

Each participant completed a total of 120 trials, including trials with different sample array sizes (two, four, and six), which were presented in a random order (40 trials for each sample array size). All AVG experts, as well as 30 of the 33 non-experts, participated in the visual WM task.

### Magnetic Resonance Imaging Acquisition

A 3-Tesla whole-body MRI scanner (Siemens Magnetom Trio; Erlangen, Germany) was used for the experiment. T1-weighted three-dimensional structural images covering the entire brain were acquired with a magnetization-prepared, rapid-gradient, echo sequence (repetition time; TR = 2,000 ms, echo time; TE = 4.38 ms, flip angle; FA = 8°, field of view; FOV = 192 mm2, inversion time; TI = 990 ms, matrix = 176×192×160, voxel size = 1×1×1 mm3, 160 axial slices). T1 images were obtained from all participants.

### Voxel-Based Morphometry Analysis

VBM analysis was conducted to quantify differences in GM volumes between AVG experts and non-experts [26]. T1-weighted volumetric images were analyzed using the SPM8 (http://www.fil.ion.ucl.ac.uk/spm) and VBM8 toolboxes (http://dbm.neuro.uni-jena.de/vbm) implemented in Matlab R2011a (Math Works, Natick, MA, USA).

We used the VBM procedure recommended by the VBM 8 toolbox manual for analysis. Prior to statistical analysis, we took the following spatial pre-processing steps: [(1)] checking for scanner artifacts and gross anatomical abnormalities for each participant; [(2)] setting the image origin to the anterior commissure; [(3)] intra-participant bias correction for MRI inhomogeneity due to gradient distortions, [(4)] segmentation of different tissue classes, [(5)] linear (affine) and nonlinear spatial normalization using Diffeomorphic Anatomical Registration using Exponentiated Lie algebra (DARTEL) [41] template in standard space provided by the Montreal Neurological Institute (MNI), and [(6)] modulation of different tissue segments by nonlinear normalization parameters to correct for individual differences in brain size. We used the DARTEL template, which was derived from 550 healthy participants in the IXI-database (http://www.braindevelopment.org). In the normalization process, voxel size was re-sampled from 1×1×1 mm to 1.5×1.5×1.5 mm[3]. The segmentation procedure was refined by accounting for partial volume effects [42], applying adaptive maximum *a posteriori* estimations [43], and applying a hidden Markov random-field model [44]. Finally, normalized GM segments were smoothed using a 10-mm full-width half-maximum (FWHM) Gaussian kernel. Following the preprocessing steps, smoothed, modulated, normalized data were obtained for statistical analysis.

For statistical analysis, pre-processed GM image segments from each group were entered into a voxel-wise two-sample *t*-test analysis in SPM8. Participant age was included as a nuisance covariate. An absolute threshold mask of 0.20 was used to avoid possible edge effects around the border between GM and white matter. The statistical threshold was set to *p*<0.05 at the voxel level, correcting for family-wise error (FWE) based on Gaussian random field theory [45]. For visualization purposes, we used the more liberal threshold of p<0.001, uncorrected for multiple comparisons. We also performed a region of interest (ROI) analysis according to previous reports of increased GM volume in the striatum of video-game players [24], [46], [47]. The target ROI was the small spherical region (r = 15 mm) around the left striatum (MNI coordinate x = −9, y = 8, z = 4) [24].

In the present study, group size was unbalanced between the groups. To investigate the robustness of the SPM result, we conducted a separate non-parametric analysis [48], [49] using the statistical non-parametric mapping (SnPM) toolbox (http://www2.warwick.ac.uk/fac/sci/statistics/staff/academic-research/nichols/software/snpm/). SnPM uses the general linear model (GLM) to construct pseudo t-statistic images, which are then assessed for significance using a standard non-parametric multiple comparisons procedure based on randomization [50] and permutation [51] tests. We used the conventional SnPM procedure recommended by the SnPM toolbox manual for analysis, and tested group differences against 1000 random permutations. We used the same statistical threshold as in the SPM analysis.

## Results

### Visual Working Memory Task

We evaluated performance differences (percentage of correct responses) in the visual WM task between AVG experts and non-experts using a two-way repeated measures analysis of variance (ANOVA) with GROUP (expert or non-expert) and ARRAY SIZE (two, four, or six) as factors (Fig. 1B). The GROUP×ARRAY SIZE interaction (F_(2,90)_ = 2.92, *p* = 0.06) was marginally significant, and the main effect of ARRAY SIZE (F_(2,90)_ = 120.23, *p*<0.001) and GROUP (F[_(1], [45)_] = 5.64, *p* = 0.02) was significant. AVG experts (87.7%) performed better than non-experts (82.0%) in the visual WM task. A previous study reported that AVG experts far out-performed non-gamers in the same visual WM task, especially when exposed to a large set size (size six) condition [15]. To address this, we compared group performance for different set sizes separately. The planned comparisons between the percentage of correct responses in the AVG expert and non-expert groups revealed that the percentage of correct responses from array size 6 in AVG experts was significantly greater than that in non-experts (*p*<0.05 with Bonferroni correction for multiple comparisons).

### Voxel-Based Morphometry Analysis

**Figure 2:**
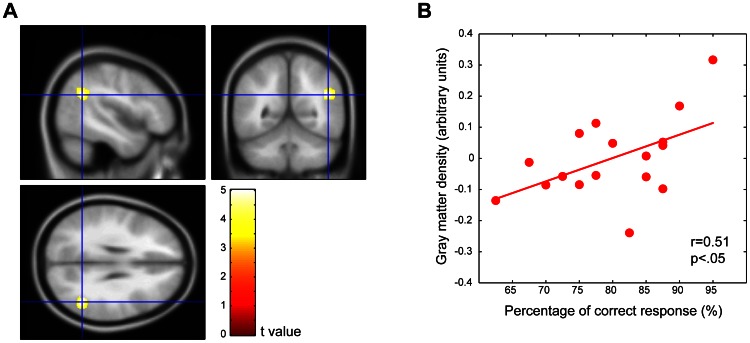
VBM analysis. A: Group activation superimposed on a standardized anatomical image. Brain region exhibits larger local GM volumes in the right IPL in AVG experts compared with non-experts (FWE-corrected *p*<0.05). The statistical threshold of the displayed image was set to a *P*-value of 0.001, which was uncorrected for multiple comparisons for display purposes only. B: A scatter-plot portraying the relationship between GM volumes in the right IPL (vertical axis) and the percentage of correct responses (horizontal axis) in the visual WM task with an array size of 6 in AVG experts. The circle represents individual values for AVG experts, and the line indicates the linear fit for these data. A significant positive correlation was observed (*p*<0.05).

Figure 2A shows the results from the VBM SPM analysis. AVG experts had significantly higher regional GM volume in the right inferior parietal lobule (IPL) compared with non-experts (peak MNI coordinate x = 47, y = −54, z = 27; t = 4.99, FWE-corrected *p* = 0.030, continuous cluster size above statistical threshold  = 22 voxels). In the whole brain analysis, the right IPL was the only area showing a significant increase in AVG experts. In contrast, non-experts exhibited no significant increase in regional GM volumes in any regions compared with AVG experts. To address the possibility that the difference in sample size between the two groups had biased the present result, we performed the SPM analysis with equal group sizes by randomly excluding 16 non-experts. We found a very similar pattern of GM difference to that obtained by our initial analysis, suggesting that the difference in sample size had not biased the present result.

We used SnPM analysis to examine the robustness of the SPM result. SnPM analysis revealed that VG experts had significantly larger regional GM volume in the right IPL compared with non-experts (peak MNI coordinate x = 47, y = −55, z = 27; pseudo t = 4.99, FWE-corrected *p* = 0.026, continuous cluster size above statistical threshold  = 38 voxels). The SnPM result supported the robustness of the present SPM result.

To investigate the relationship between the larger GM volume in the right IPL and superior visual WM task performance in AVG experts, we conducted a correlation analysis (Pearson's *r*; Fig. 2B). A significant positive correlation was found between right IPL GM volume and expert visual WM task performance with an array size of six (*r* = 0.512, *p* = 0.036), but not with an array size of two or four. A Smirnov-Grubbs test detected no significant outliers in the data used for correlation analyses. This correlation was not observed in non-experts (r = −.02, p = .92).

To further assess the relationship between structural changes in the IPL and game training in the experts group, we conducted a correlation analysis between the GM volume in the right IPL and (i) the years of video game play, (ii) the age of video game play commencement, and (iii) the level of video game performance (ranking in *The Super Battle Opera Tournament*, n = 16) in the expert group. No significant correlations resulted from any of these analyses (years of video-game play, r = −.13, *p* = .63; age of commencement, r = −.02, *p* = .94; level of performance, r = −.13, *p* = .62).

A ROI-based analysis revealed a significant difference in GM volume in the left caudate nucleus (peak MNI coordinate x = −11, y = 0, z = 16; t = 3.77, FWE-corrected p = 0.013, continuous cluster size above statistical threshold  = 49 voxels). This finding is consistent with the results of several previous VBM studies that reported greater GM volume in the striatum of video-game players [24], [46], [47].

## Discussion

The present study revealed two major novel findings. First, VBM analysis revealed significantly larger GM volumes in the right PPC, especially in the right IPL, of AVG experts compared with non-experts. Second, larger GM volume in the right PPC was positively correlated with superior visual WM performance in AVG experts.

Previous studies have demonstrated a role for the right PPC in visuospatial function [33]–[39]. Our VBM findings are consistent with those of previous behavioral studies that report superior visuospatial task performance in AVG players [3]–[8], [12]–[14], [16]. Our results suggest that larger right PPC volume may constitute the neural basis for increased visual performance in AVG players. We found that visual WM performance was positively correlated with larger GM volume in the right PPC in AVG experts for an array size of six, but not two or four. This positive correlation indicates that larger GM volume in the right PPC is associated with increased WM performance (especially with an array size of six) in AVG experts. Previous studies have demonstrated that visual WM performance on tasks with larger set sizes reflects how efficiently participants select items to be remembered, as opposed to how many items they can hold in their visual WM [52]–[55]. Therefore, it is likely that the observed increase in GM volume in the PPC is more closely related to the efficiency of attentional control in the visual WM, rather than the number of items participants can hold in their visual WM. This view is consistent with previous reports of superior performance on spatial attention tasks in AVG players [3]–[6], [8]. We speculate that more efficient attentional control, which is likely mediated by a neural network that includes the right PPC, might be responsible for increased WM performance in AVG experts [53], [54], [56].

While GM volume in the PPC was positively correlated with the visual WM task performance at set size six in the expert group, this relationship was not found in the non-expert group. In the visual WM task, non-experts exhibited poor performance compared to experts for an array size of six. We speculate that GM volume in the PPC may not increase until efficiency of attentional control reaches a certain level (or vice versa).

Recent electrophysiological and neuroimaging studies have generated a neurophysiological index of superior attentional processes (*e.g*., larger P300 component amplitude and reduced functional MRI activity in the frontoparietal network) in AVG players [22], [23], [25]. In the present study, an increase in region-specific GM volume was observed in the PPC of AVG experts. The PPC is an essential cortical structure for visuospatial function, including attentional processes [34], [35]. Therefore, the present VBM results may represent a neuroanatomical index of superior visuospatial processes in AVG players, consistent with neurophysiological findings. The present results provide evidence for region-specific structural differences in AVG experts compared with non-experts, and provide more precise anatomical localization compared to other functional neuroimaging studies [22], [23], [25].

Previous VBM studies have found region-specific differences in GM volume related to the development of new skills, such as golf, juggling, or studying for medical examinations [57]–[61]. These findings suggest that the increased GM volume in the right PPC observed in the present study might be the result of extensive and long-term AVG training. For instance, that behavioral differences between AVG players and non-players are due to AVG experience and not to other pre-existing differences has been repeatedly demonstrated [3], [5], [7], [8], [10], [11], [62], [63]. These results suggest that AVG players might acquire superior visual skill associated with increased GM volume in the right PPC as their expertise increases.

Alternatively, it is possible that individuals with a high degree of AVG experience possess inherently greater visuospatial cognitive function and have larger right PPC volume. There are two reasons why is important to consider this possibility in light of the present findings: First, participants in previous studies were typically college students who played video games quite often, or who received training with the video games as part of the experiments [3], [5], [10]. In contrast, the participants in the present study were professional game players who were at or near the top of the world rankings. Therefore, it is reasonable to consider that individuals who have achieved a high level of video-game performance may possess inherently greater visuospatial cognition and may have larger GM volumes in the PPC. Alternatively, an interaction between inherent ability and extensive training may have induced the observed neuroanatomical changes. Second, in the present study, the larger GM volume in the right PPC was not significantly correlated with video game experience or performance level. This negative result implies that the larger GM volume in the expert group might be independent of video game experience. The present study did not seek to demonstrate a causal link between AVG playing and changes in regional GM volume, but instead aimed to test region-specific differences in neuroanatomical structure between AVG experts and non-experts. Future longitudinal studies would be necessary to determine causality.

While many previous studies examined individuals who play shooter games (e.g., [3]), we examined players of a fighting video game, *Guilty Gear*. Shooter games generally contain a challenging attentional-control component, whereas *Guilty Gear* primarily requires visuo-motor skill, in addition to fine attentional control. It is unclear how these differences in gaming sub-genres may contribute to the present VBM finding. However, it is likely that the different cognitive and motor requirements among the AVG sub-genres may have different effects on cognitive and motor function [3], and may be linked to different types of neuroanatomical development in a genre-specific manner. Further systematic investigations are necessary to reveal genre-specific effects on brain and behavior.

In this study, we did not measure the IQ of the participants. This is a limitation of the present study. However, we did match educational level between the groups. We speculate that between-group differences in general IQ score would be minimal in the present experiment.

In summary, the present study examined region-specific differences in GM volume in AVG experts compared with non-experts. VBM analysis revealed larger GM volume in the right PPC of AVG experts, which was positively correlated with superior performance in a visual WM task. Thus, this study demonstrates that AVG players exhibit differences in brain structure compared with non-players, suggesting that structural differences may be partially responsible for superior visual performance in AVG players.
